# ﻿The Tubulifera (Hexapoda, Thysanoptera) of the Maltese Islands

**DOI:** 10.3897/zookeys.1180.107065

**Published:** 2023-09-21

**Authors:** Godwin Degabriele, Adriano Cavalleri, Arturo Goldarazena, David Mifsud

**Affiliations:** 1 Institute of Earth Systems, Rural Sciences and Food Systems, University of Malta, MSD 2080 Msida, Malta University of Malta Msida Malta; 2 Biological Sciences Institute, Carreiros Campus, Federal University of Rio Grande, Rio Grande 96203-900, Brazil Federal University of Rio Grande Rio Grande Brazil; 3 National Museum of Natural Sciences-CSIC, Department of Biodiversity and Evolutionary Biology National Reference Laboratory for Nematodes and Arthropods, C/Serrano Duplicado, CP 28006 Madrid, Spain National Museum of Natural Sciences-CSIC, Department of Biodiversity and Evolutionary Biology National Reference Laboratory for Nematodes and Arthropods Madrid Spain

**Keywords:** Chorotypes, feeding preferences, identification keys, Malta, Mediterranean Sea, taxonomy, thrips

## Abstract

This work records the presence of 13 species of tubuliferan thrips from the Maltese Islands. Eleven of these species, namely *Bolothripsdentipes*, *B.insularis*, *Priesneriellamavromoustakisi*, *Gynaikothripsuzeli*, *Haplothripsacanthoscelis*, *H.aculeatus*, *H.setiger*, *H.tritici*, *Karnyothripsflavipes*, *Liothripsreuteri* and *Neoheegeriadalmatica* are new records for the Maltese Islands. Two species: *Gynaikothripsficorum* and *Karnyothripsflavipes* can be described as subcosmopolitan in distribution, another three species: *Haplothripsaculeatus*, *H.setiger* and *H.tritici* are distributed across the Holarctic and Palaearctic regions, while a further seven: *Bolothripsdentipes*, *B.insularis*, *Haplothripsacanthoscelis*, *Liothripsoleae*, *L.reuteri*, *Neoheegeriadalmatica* and *Priesneriellamavromoustakisi* have a European and/or Mediterranean distribution. *Gynaikothripsficorum* and *G.uzeli* are considered as alien species. A key to the Tubulifera of the Maltese Islands as well as chorological data for these recorded species are provided in this work.

## ﻿Introduction

Thrips are insects that belong to the order Thysanoptera, a relatively small group of insects which includes around 6400 described species worldwide. This order is divided into the suborders Terebrantia and Tubulifera. Thrips species under these two groups differ physiologically and behaviourally.

**Figure 1.**
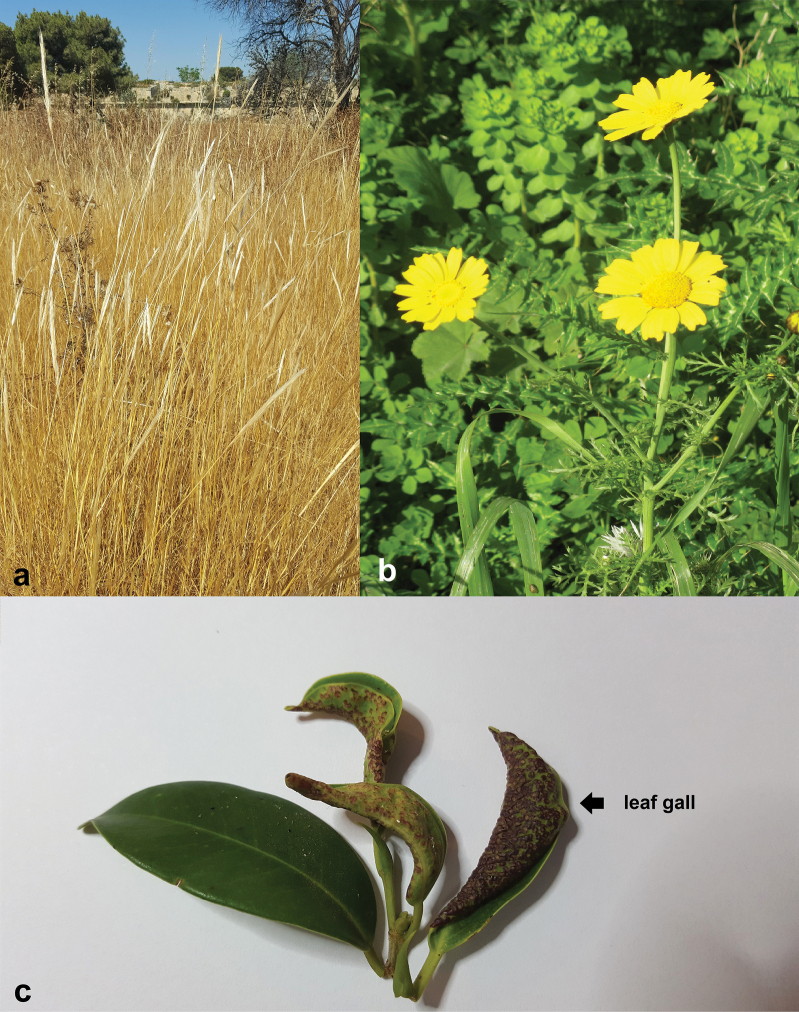
Typical habitats of tubuliferan thrips in the Maltese Islands **a** bases of plants, such as grasses (*Gastridiumventricosum*) **b** flowers, such as those of the Asteraceae (*Glebioniscoronaria*) **c** galls induced on leaves (*Ficusmicrocarpa*).

The Tubulifera includes one large family, Phlaeothripidae, that comprises over 3600 extant and 20 extinct species (ThripsWiki 2023) and is subdivided into two subfamilies, the Idolothripinae (734 described species) and the Phlaeothripinae (2877 described species) (ThripsWiki 2023). All Idolothripinae feed on fungal spores and a large number of species of Phlaeothripinae also feed on fungal hyphae (Mound and Tree 2018). These mycophagous species typically occur at the base of dried plants, amongst dead and decaying twigs and branches as well as in leaf litter (Fig. 1a). Other tubuliferans such as some *Haplothrips* and *Karnyothrips* are known to feed on pollen and can be abundant in inflorescences such as those of Asteraceae (Fig. 1b) (Lewis 1973).

Many Phlaeothripinae also feed on green leaves and at least 300 species can induce galls on the leaves of their host-plants (Fig. 1c) (Crespi et al. 1998; Mound and Morris 2005). These species, for example, those of the genus *Gynaikothrips* and some others of the genus *Liothrips*, tend to live in aggregations within the galls. The predatory behavior evolved many times among Phlaeothripinae, such as in some *Leptothrips* and *Karnyothrips* species, which are reported to feed on smaller arthropods (Mound and Marullo 1996; Cavalleri et al. 2016).

**Figure 2.**
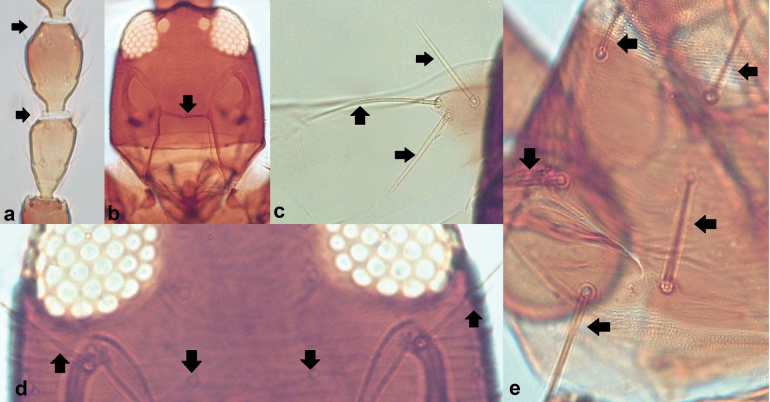
Useful distinguishing features in Tubulifera**a** sensoria on antennal segments III–IV **b** maxillary bridge in the head capsule; body setae (**c-e**) **c** basal setae on the forewing **d** post-ocular setae **e** pronotal setae.

Different species of Tubulifera can be rather difficult to recognize at species level and several diagnostic characters include minute structures that require some experience by researchers for a correct interpretation. Some of these features include: the number of sensoria on antennal segments III and IV (Fig. 2a); the presence or absence of a maxillary bridge inside the head capsule (Fig. 2b); the length and shape of some of the wing and body setae (Fig. 2c–e).

The geography, climate, natural habitats and the impact of the high population density on the natural environment of the Maltese Islands have already been described in the work related to the study of the Terebrantia of the Maltese Islands (Degabriele et al. 2023).

Previous literature on the Tubulifera of the Maltese Islands consists of two papers that record the presence of two species. The first species is *Liothripsoleae* Costa (Haber and Mifsud 2007), considered a pest of olive crops and the second is *Gynaikothripsficorum* (Marchal) (Mifsud et al. 2012; de Jong et al. 2014) which induces galls on the leaves of ornamental *Ficus* plants.

This current study was therefore carried out to record and document the biodiversity of the tubuliferan thrips found in the Maltese Islands. It also provides an illustrated dichotomous key for these species, as well as an account of their feeding habits and the geographical distribution.

## ﻿Material and method

This work forms part of a study on the biodiversity of Thysanoptera of the Maltese Islands conducted between 2015 and 2022. The terebrantian species recorded in this study have already been published (Degabriele et al. 2023).

Tubuliferan thrips were collected from 41 different locations which featured different habitats in Malta and Gozo. The habitats have been described in detail in the study on Terebrantia (Degabriele et al. 2023). Methods used for collecting specimens, selection of locations and plants, as well as methods to slide-mount material and identification all follow Degabriele et al. (2023).

All material collected was carefully examined to differentiate as much as possible between different species. One-hundred and twenty specimens were slide mounted to be further studied under compound microscopy using a Leica DM3000 microscope with DIC/ Phase contrast microscope illumination and fitted with a Leica ICC50 camera and a Leica DVM6 otical microscope. A number of specimens from the remaining wet collection, which were preserved in AGA mixture made up of 10 parts of 60% ethyl alcohol, one part of glycerine and one part of glacial acetic acid were also examined. This described material forms part of the private collection of Godwin Degabriele (GD). Another five specimens from the private collection of David Mifsud (DM), which were collected in the late 1990s to the mid-2000s were also included in this work. Voucher specimens will be deposited at the Museo de Ciencias Naturales, Madrid, Spain.

Abbreviations used in the “Material examined” sections listed below include the following: Godwin Degabriele (GD); Charles Farrugia (CF); David Mifsud (DM); slide mounted specimens (sm); specimens conserved in AGA solution (aga). Other abbreviations include: seta pair I on abdominal tergite IX found the mid-line (S_1_); thrips which represent new record for the Maltese Islands (†).

## ﻿Results

The current study has provided an account of the tubuliferan thrips that occur in the Maltese Islands. These consist of 13 species belonging to one family and seven genera. Eleven species are new records to the Maltese Islands.

### ﻿Key to Tubulifera of the Maltese Islands

**Table:** 

1	Abdominal segment X conical and with a longitudinal split in females (Fig. 3a), sometimes rounded in males; fore wings with one or two longitudinal veins (Fig. 3c); all wings covered with microtrichia (Fig. 3c); females with external ovipositor	**Terebrantia**
–	Abdominal segment X tubular in both sexes (Fig. 3b); fore wings with no veins (Fig. 3d); all wings with smooth surface (Fig. 3d); females lacking external ovipositor	**2** (**Tubulifera)**
2	Maxillary stylets broad, usually more than 5 µm in width throughout their length (Fig. 4a); generally with fungal spores visible in the digestive tube (Fig. 4c)	**3** (**Idolothripinae)**
–	Maxillary stylets slender, usually 2–3 µm in width (Fig. 4b); fungal spores never visible in the digestive tube (Fig. 4d)	**5** (**Phlaeothripinae)**
3	Antennae seven-segmented, with segments VI and VII broadly joined (Fig. 5a); abdominal segment X yellow (Fig. 5c)	***Priesneriellamavromoustakisi* (Crawford)**
–	Antennae eight-segmented with all segments distinct from each other (Fig. 4b); abdominal segment X brown (Fig. 5d)	**4**
4	Eyes about 1.3 times longer ventrally than dorsally (sometimes less) (Fig. 6a); abdominal segment X more than 2.0 times as long as its basal width (Fig. 6c); S_1_ on abdominal segment IX shorter than segment X (Fig. 6c); body colour dark brown (Fig. 6c)	***Bolothripsdentipes* (Reuter)**
–	Ventral length of compound eyes longer, at least 1.6 times the dorsal length (Fig. 6b); abdominal segment X shorter, less than 2.0 times its basal width (Fig. 6d); S_1_ on abdominal segment IX longer than segment X; body colour light brown (Fig. 6d)	***Bolothripsinsularis* (Bagnall)**
5	Fore wings constricted medially (Fig. 7a); head with maxillary bridge present (Fig. 7c); prosternum with basantra (Fig. 7e); antennal segment III with one or two sense cones; segment IV with four sense cones	**6**
–	Fore wings not constricted medially (Fig. 7b); maxillary bridge absent (Fig. 7d); prosternum with no basantra (Fig. 7f); antennal segment III with three sense cones; segment IV with two sense cones	**11**
6	Antennal segment IV with three sense cones (Fig. 8a); basantra as long as broad (Fig. 8c)	***Karnyothripsflavipes* (Jones)**
–	Antennal segment IV with four sense cones (Fig. 8b); basantra broader than long (Fig. 8d)	**7**
7	Antennal segment III with three sense cones (Fig. 9a); fore wing with 12–18 duplicate cilia (Fig. 9c)	***Neoheegeriadalmatica* Schmutz**
–	Antennal segment III with one to two sense cones (Fig. 9b): fore wing with not more than nine duplicate cilia (Fig. 9d)	**8 (*Haplothrips* Amyot & Serville)**
8	Antennal segment III prominently asymmetrical and with one sense cone (Fig. 10a); pronotum with prominent postero-angular and epimeral setae, other setae being as long as discal setae (Fig. 10c); ommatidia occasionally with internal red pigment (Fig. 10e)	***Haplothripsaculeatus* (Fabricius)**
–	Antennal segment III symmetrical and with two sense cones (Fig. 10b); pronotum with all five pairs of setae well developed (Fig. 10d); compound eyes never with red pigment	**9**
9	Head with post-ocular and pronotal setae with a capitate tip (Fig. 11a)	***Haplothripsacanthoscelis* (Karny)**
–	Post-ocular and pronotal setae with a pointed or blunt tip (Fig. 11b)	**10**
10	Fore wing tip cilia barbed (Fig. 12a); post-ocular setae with pointed tips.	***Haplothripssetiger* Priesner**
–	Fore wing tip cilia smooth (Fig. 12b); post-ocular setae with blunt tips.	***Haplothripstritici* (Kurdjimov)**
11	Mouth cone rounded at tip (Fig. 13a); pronotal sculpture reticulate (Fig. 13c); antero-angular, antero-marginal and mid-lateral setae as long as or very slightly longer than discal setae (Fig. 13c); both sexes with a small fore tarsal tooth (Fig. 13e, f). Develop on *Ficus*	**12 (*Gynaikothrips* Zimmermann)**
–	Mouth cone long and pointed (Fig. 13b); pronotal sculpture striate and not always distinct (Fig. 13d); antero-angular, antero-marginal and mid-lateral setae longer than discal setae (Fig. 13d); fore tarsal tooth absent in both sexes (Fig. 13g, h). Develop on different plants other than *Ficus*	**13 (*Liothrips* Uzel)**
12	Head with with post-ocular setae i 0.5–1 times as long as post-ocular setae ii and post-ocular setae ii not overlapping posterior margin of compound eye (Fig. 14a); pronotum with postero-angular setae scarcely developed, much shorter than epimeral setae (Fig. 14c)	***Gynaikothripsficorum* (Marchal)**
–	Head with post-ocular setae i 0.3–0.5 times as long as post-ocular setae ii and post-ocular setae ii considerably overlapping posterior margin of compound eye (Fig. 14b); pronotum with postero-angulars and epimerals almost equal in length (Fig. 14d)	***Gynaikothripsuzeli* (Zimmermann)**
13	Fore wing with around 17 duplicate cilia (Fig. 15a); lateral setae on cheeks longer than discal setae (Fig. 15c); fore tibiae and tarsi yellow (Fig. 15e). Develops on *Oleaeuropea*	***Liothripsoleae* (Costa)**
–	Fore wing with less than seven duplicate cilia (Fig. 15b); lateral setae on cheeks not longer than discal setae (Fig. 15d); fore tibiae and tarsi brown (Fig. 15f). Develops on *Tamarix*	***Liothripsreuteri* (Bagnall)**

**Figure 3.**
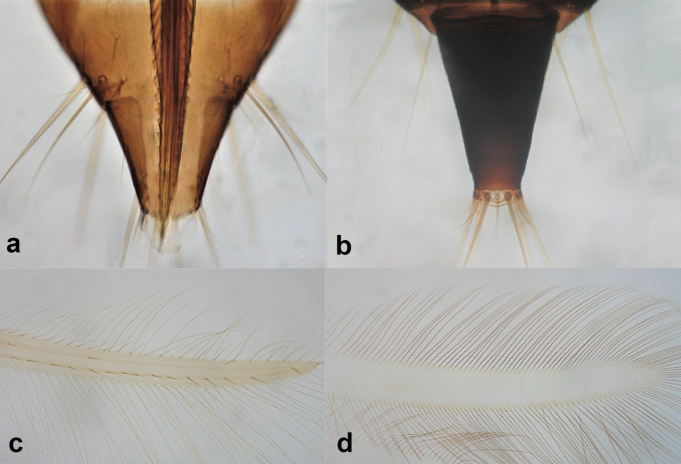
Abdominal segment X (**a, b**) **a** conical **b** tubular; fore wings (**c, d**) **c** with longitudinal veins **d** with no veins.

**Figure 4.**
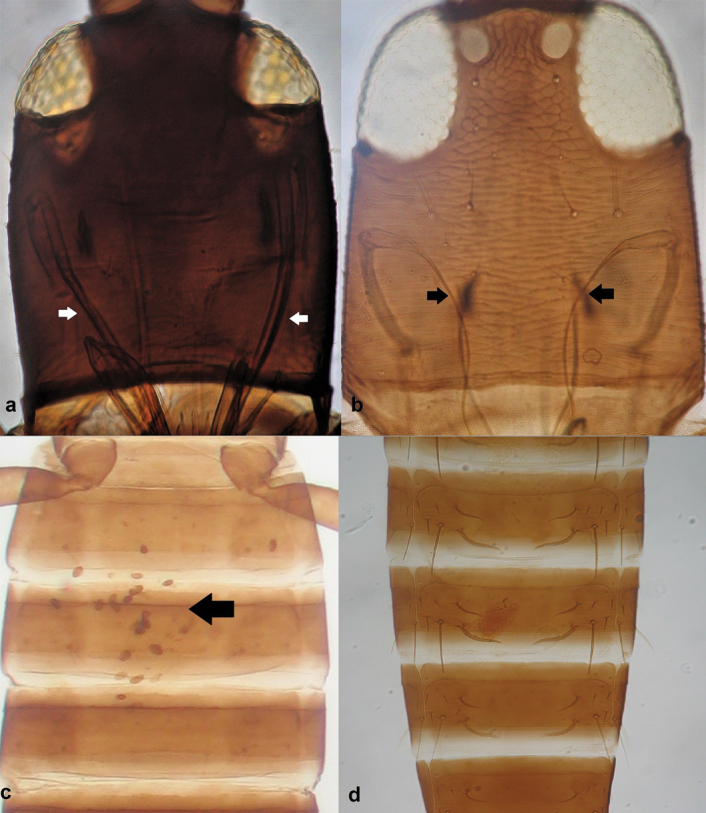
Head (**a, b**) **a** with maxillary stylets broader than 5 µm throughout their length **b** with maxillary stylets around 2–3 µm throughout their length; habitus (**c, d**) **c** with fungal spores present in digestive tube **d** with no fungal spores in digestive tube.

**Figure 5.**
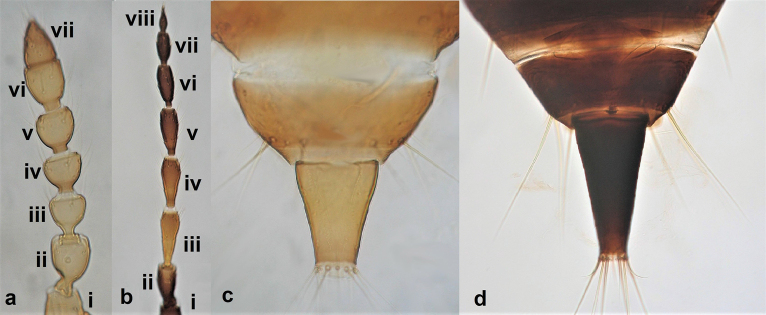
Antennae (**a, b**) **a** 7-segmented **b** 8-segmented; abdominal segment X (**c, d**) **c** pale-coloured **d** dark coloured.

**Figure 6.**
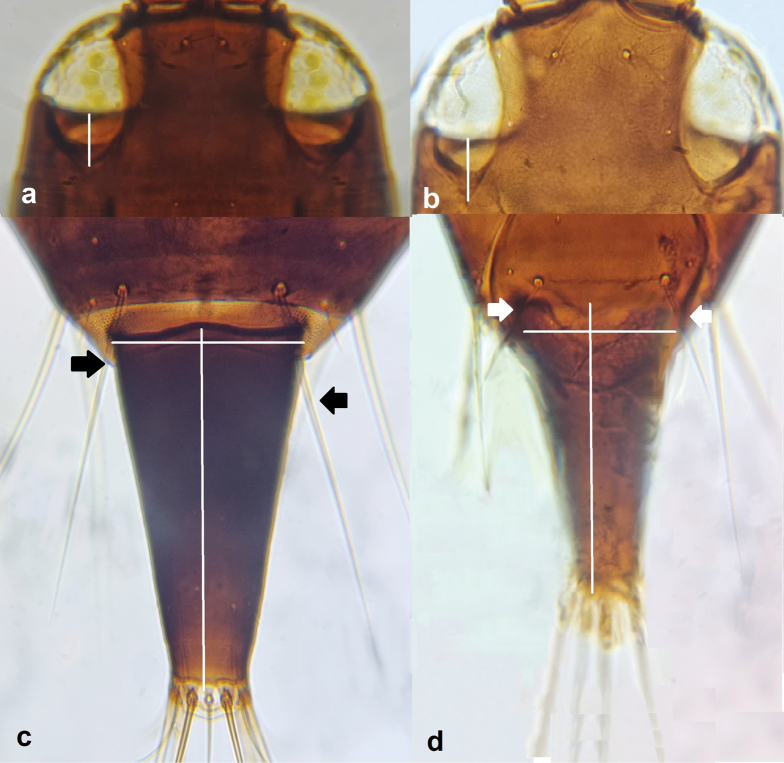
Upper section of head showing compound eyes (**a, b**) **a** with ventral length of compound eyes about 1.3 times the dorsal length **b** with ventral length of compound eyes at least 1.6 times the dorsal length; abdominal segments (**c, d**) **c** dark brown and with setae S_1_ on abdominal segment IX shorter than segment X **d** light brown and with setae S_1_ on abdominal segment IX longer than segment X.

**Figure 7.**
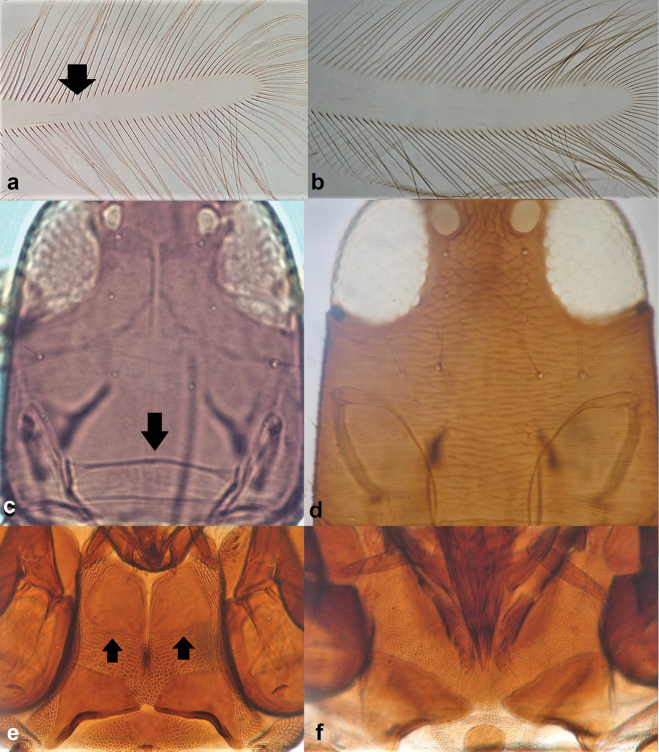
Fore wing (**a, b**) **a** showing medial constriction **b** with no medial constriction; head (**c, d**) **c** with maxillary bridge **d** with no maxillary bridge; prosternum (**e, f**) **e** with basantra **f** with no basantra.

**Figure 8.**
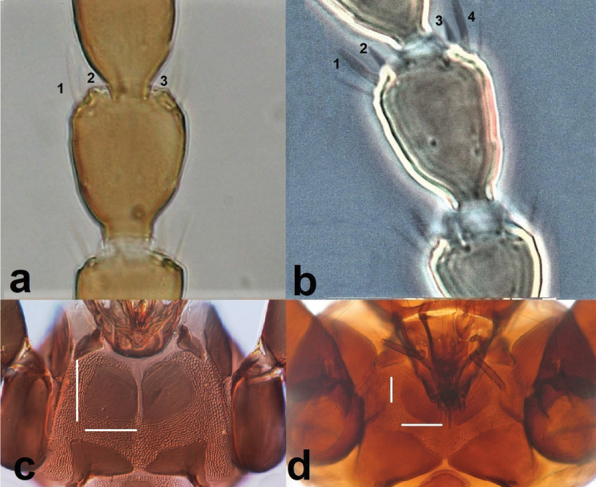
Antennal segment IV (**a, b**) **a** with three sense cones **b** with four sense cones; pronotum (**c, d**) **c** with basantra as long as broad **d** with basantra broader than long.

**Figure 9.**
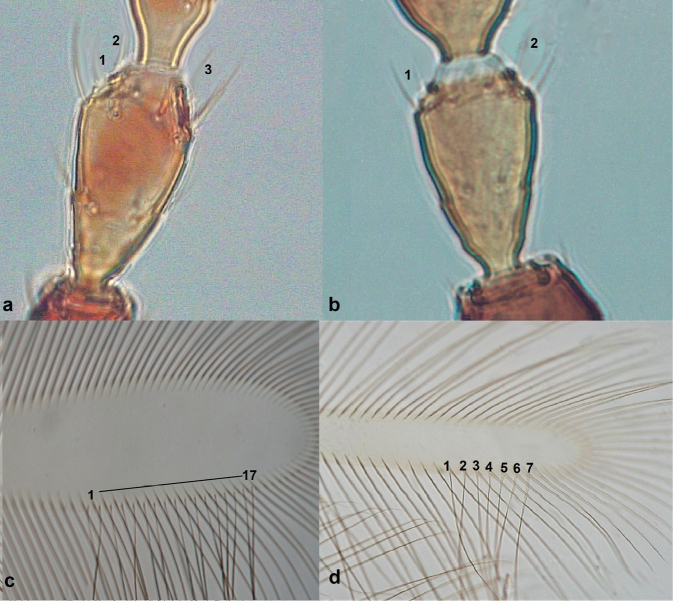
Antennal segment III (**a, b**) **a** with three sense cones **b** with two sense cones; fore wing (**c, d**) **c** with 17 duplicate cilia **d** with seven duplicate cilia.

**Figure 10.**
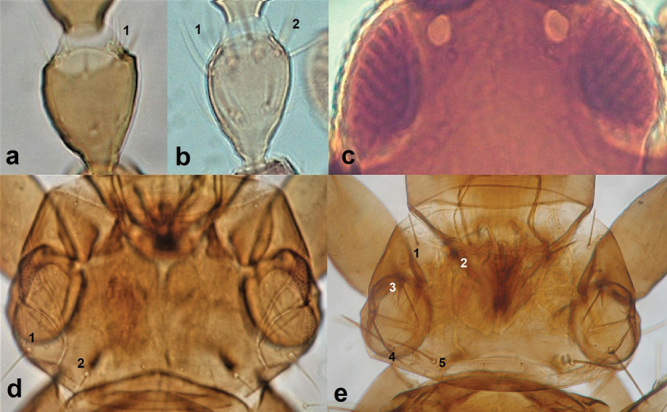
Antennal segment III (**a, b**) **a** one sense cone **b** two sense cones **c** compound eyes showing red internal pigment; pronotum (**d, e**) **d** with two pairs of prominent setae **e** with five pairs of prominent setae.

**Figure 11.**
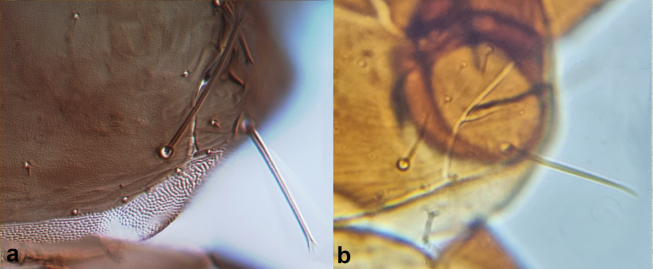
Postero-angular and epimeral setae on pronotum **a** capitate **b** pointed.

**Figure 12.**
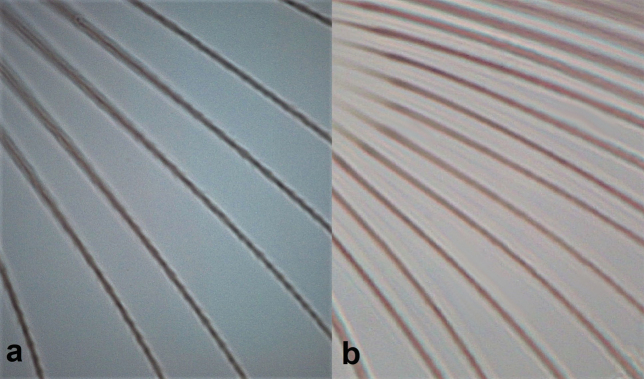
Fore wing tip cilia **a** barbed **b** smooth.

**Figure 13.**
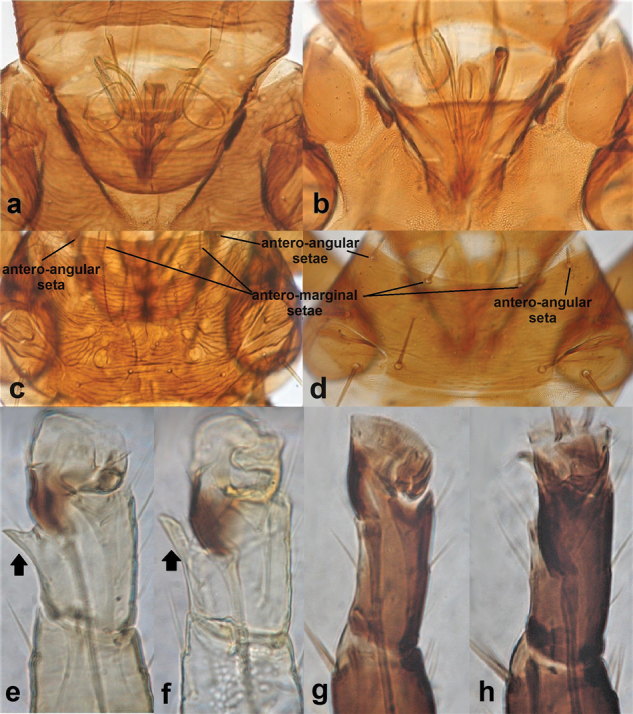
Mouth cone (**a, b**) **a** rounded **b** pointed; pronotum (**c, d**) **c** with sculpture and with antero-angular, antero-marginal and medial setae as long as discal setae **d** without a distinct sculpture and with antero-angular, antero-marginal. and medial setae as longer than discal setae; fore tarsus (**e–h**) **e, f** toothed **g, h** with no tooth.

**Figure 14.**
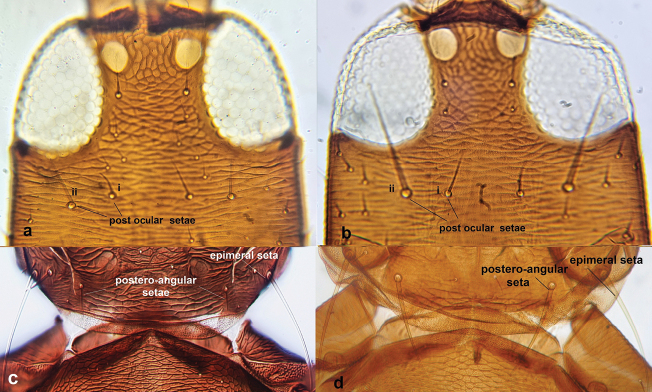
Head (**a, b**) **a** with post-ocular setae i 0.5–1 times as long as post-ocular setae ii and post-ocular setae II not overlapping posterior margin of compound eye **b** with post-ocular setae i 0.3–0.5 times as long as post-ocular setae ii and post-ocular setae ii considerably overlapping posterior margin of compound eye; pronotum (**c, d**) **c** with postero-angular setae much shorter than epimeral setae **d** with postero-angular and epimeral setae almost equal in length.

**Figure 15.**
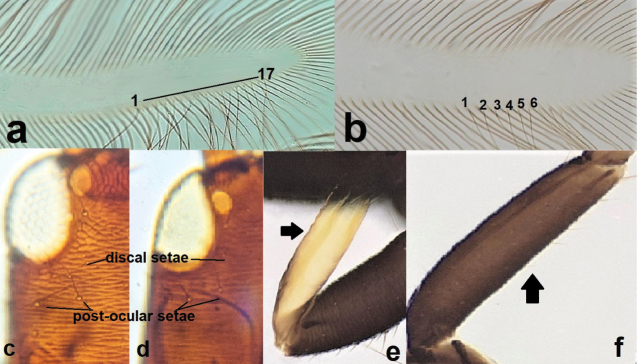
Fore wing (**a, b**) **a** with 17 duplicate cilia **b** with six duplicate cilia; head showing lateral seta on cheek (**c, d**) **c** longer than discal setae **d** not longer than discal setae; fore tibia (**e, f**) **e** yellow **f** brown.

### ﻿Species catalogue

**Family Phlaeothripidae**

#### ﻿Subfamily Idolothripinae

***Bolothripsdentipes* (Reuter, 1880)** †

**Material examined. Malta**: Il-Ballut l/o M’Xlokk, 03.v.2017, 5 ♀♀ (sm, aga) and 2 ♂♂ (sm, aga) on *Arthrocnemummacrostachyum*, GD leg.; Ta’ Sabbara woodland, 19.v.2017, 5 ♀♀ (sm, aga) on dried *Gastridiumventricosum*, GD leg.

**Body length.** ♀: 2360–2800 µm; ♂: 2300 µm.

**Wing type.** Both sexes are apterous.

This species is spore-feeding and occurs mainly at the base of Poaceae (Mound et al. 1976; Mound et al. 2018). In the Maltese Islands, both larvae and adults were collected from the base of dried *Gastridiumventricosum* (Poaceae) as well as from the base of the halophytic caryophyllate *Arthrocnemummacrostachyum* (Amaranthaceae). This suggests that fungi associated with these plants are the food resource for *B.dentipes*. Analysis of spores visible in the gut of mounted specimens of *B.dentipes* reveal that the fungus is possibly a species of *Psathyrella* (Agaricales) (S. Mifsud, pers. comm., 20.05.2022). *Bolothripsdentipes* is widely distributed in Europe (Mound et al. 1976).

***Bolothripsinsularis* (Bagnall, 1914)** †

**Material examined. Malta**: Wied Speranza, 26.iv.2016, 1 ♂ (sm) from *Convolvuluselegantissimus*, GD leg.

**Body length.** ♀: no records; ♂: 2270 µm.

**Wing type.** ♀: no records; ♂: apterous.

Like the congener mentioned above, *Bolothripsinsularis* is also a spore-feeding species which lives at the base of plants, mainly grasses (zur Strassen 1986; Marullo 1999). The single specimen collected from the Maltese Islands was found in the flowers of *Convolvuluselegantissimus* (Convolvulaceae). *Bolothripsinsularis* is restricted to the Mediterranean area between the Canary Islands and Syria (Mound 1974).

***Priesneriellamavromoustakisi* (Crawford, 1948)** †

**Material examined. Malta**: Xrobb l-Għagin, 24.ii.2016, 1 ♀ (sm) on *Hyparrheniahirta*, GD leg.; Wied Għollieqa, 16.iv.2018. 1 ♀ (sm) on *Ornithogalumarabicum*, GD leg.

**Body length.** ♀: 1680–1800 µm; ♂: no records.

**Wing type.** ♀: apterous; ♂: no records.

This species is typically found in lawns and on tree barks (Priesner 1964) and feeds on fungal spores (Marullo and De Grazia 2013). Males of this species were not recorded here, but they are known to exhibit a large tooth on the fore tarsus (Priesner 1964). *Priesneriellamavromoustakisi* is found across the Mediterranean basin (Mound and Marullo 1996) and has been also recorded from *Atriplexhalimus* (Amaranthaceae), *Quercuscoccifera* (Fagaceae) (zur Strassen 1986) and *Tamarixgallica* (Tamaricaceae) (Priesner 1964).

#### ﻿Subfamily Phlaeothripinae

***Gynaikothripsficorum* (Marchal, 1908)**

**Material examined. Malta**: Luqa, 25.x.1995, 1 ♀ (sm) on *Ficusmicrocarpa*, DM leg.; Msida, University of Malta grounds, 20.viii.2016, 13 ♀♀ (sm, aga) and 8 ♂♂ (sm, aga) on *Ficusmicrocarpa* leaf gall, GD leg.; Msida, Junior College grounds, 20.viii.2016, 9 ♀♀ (sm, aga) and 10 ♂♂ (sm, aga) on *Ficusmicrocarpa* leaf gall, GD leg.; Gudja, l/o Malta International Airport, 26.ix.2016, 2 ♀♀ (sm) and 1 instar larva (sm) on *Ficusmicrocarpa* leaf gall, GD leg.

**Body length.** ♀: 2400–3280 µm; ♂: 2180–2580 µm.

**Wing type.** Both sexes are macropterous.

*Gynaikothripsficorum* is a gall-inducing species described from the leaves of *Ficus* spp. (Moraceae). All stages in the life cycle of this species are found in the leaf galls of *Ficusmicrocarpa*, implying that this plant is used as a host, even though adults have also been described from *Ficuselastica* (Mound et al. 1976), *F.benjamina* (Marullo and De Grazia 2013) and *F.microcarpa* (Moritz et al. 2012). Records from the Maltese Islands match the descriptions from literature, with all life cycle stages being collected from *F.microcarpa*, where it is widely recorded as a pest forming leaf galls in Summer and Autumn. Adults were also collected from *F.benjamina*. Males of this species have been described to lack a fore tarsal tooth (Marullo and De Grazia 2013) although specimens collected from the Maltese Islands all had a small tooth on the fore tarsus. Although *G.ficorum* was first recorded locally in 2012 (Mifsud et al. 2012), it is likely that this species occurred here before that time. Indeed, *F.microcarpa* was imported to the Maltese Islands in the 1950s and 60s as part of a project designed to increase the local populations of trees in urban regions. Thus *G.ficorum* could have been accidentally introduced to the Maltese Islands with the importation of these plants. This thrips species is commonly preyed upon by the heteropteran bugs (Anthocoridae) of the genera *Orius*, *Macrotracheliella* (Moritz 2006), and *Montandoniola*, such as *M.confusa* (Funderburk et al. 2017) and *M.moraguesi* (Pluot-Sigwalt et al. 2009; Tavares et al. 2013). In the Maltese Islands, *Oriuslaevigatus*, was recorded inside or in proximity of leaf galls produced by *G.ficorum* in the current study. This heteropteran was first recorded in some locations in the Maltese Islands in 1986, and introduced in other locations in the archipelago in 1995 (Mifsud 1997). Moreover, *M.moraguesi*, was also collected with samples of *G.ficorum* (Mifsud et al. 2012), however it is not known whether its introduction was accidental with the importation of ornamental *Ficus* plants or deliberate as a pest control measure. *Montandoniolamoraguesi* was first recorded in the Maltese Islands in 1972 (Péricart 1972). The tubuliferan thrips *Androthripsramachandrai*, Karny 1926, which was originally described from India, is also a predator of *G.ficorum* (Melo et al. 2013). *Androthripsramachandrai* has not been recorded in the Maltese Islands. Apart from the damage it might inflict to *Ficus* leaves, *G.ficorum* has also been described as a possible vector of phytopatogenic bacteria and fungi (Moritz et al. 2012). This thrips species is believed to be of Asian origin (Priesner 1960), but has been imported to many parts of the world, together with its host-plant. It has been recorded from the Australian, Nearctic, Neotropical and Oriental regions, the Near East and North Africa (de Jong et al. 2014) and has also been intercepted in the US from material originating from Africa (Nickle 2003).

***Gynaikothripsuzeli* (Zimmermann, 1900)** †

**Material examined. Malta**: Msida, University of Malta grounds, 20.viii.2016, 1 ♀ (sm) on *Ficusmicrocarpa* leaf gall, 14 ♀♀ (sm, aga) and 2 instar larvae on *Ficusbenjamina* leaf gall, GD leg.; Msida, University of Malta grounds, 26.viii.2016, 1 ♂ (sm) and 5 ♀♀ (aga) on *Ficusbenjamina* leaf gall, GD leg.; Gudja, l/o Malta International Airport, 26.ix.2016, 2 ♂♂ (sm) on *Ficusmicrocarpa* leaf gall, GD leg.

**Body length.** ♀: 3200–3700 µm; ♂: 2800–2900 µm.

**Wing type.** Both sexes are macropterous.

This species induces galls on the leaves of *Ficusbenjamina* (Moraceae). Adults have also been recorded on *F.microcarpa*, *F.obtusa* and *F.pilosa* (Moritz et al. 2012). In the Maltese Islands, galls of this species containing eggs, adults and larvae were found on *F.benjamina* during Summer and Autumn. Adults were also collected from *F.microcarpa*. Specimens of *G.uzeli* collected in countries outside the Maltese Islands were described to be lacking the fore tarsal tooth absent in both sexes (Mound et al. 2019), however locally-collected male specimens did possess a small fore tooth on the fore tarsus. Studies on *G.ficorum* and *G.uzeli* reveal that these closely related species are not clearly defined due to variations in length of the post-ocular and postero-angular setae (Rodríguez-Arrieta and Retana-Salazar 2010). Moreover, it was also found that, under laboratory conditions, *G.ficorum* can also induce galls on *F.benjamina* (Tree et al. 2015), just like *G.uzeli*. Nonetheless, observations of the morphological features from local specimens matched descriptions by literature (see couplet 14 in the key) in all specimens studied and no intermediate forms were found. Moreover, Mifsud et al. (2012) in a review paper related to arthropods associated with *Ficus* species only found leaf galls on *F.microcarpa* induced by *G.ficorum*. At the time, no galls were found on *F.benjamina* despite repeated field work (Mifsud, pers. obs. 2022). The clearly defined lengths of the post-ocular and postero-marginal setae, as well as the fact that no galls were ever found on *F.benjamina* prior to 2016, when the first specimens of *G.uzeli* were recorded in the Maltese Islands suggest that *G.ficorum* and *G.uzeli* are in fact separate species. Just as in the case of *G.ficorum*, bugs of the genus *Orius* (Anthocoridae) were observed inside or in proximity of the galls induced by *G.uzeli*. The tubuliferan thrips *Androthripsramachandrai* has been described as a predator for *G.uzeli* from Syria and Cyprus (Ali 2015; Collins and Philippou 2016). *Gynaikothripsuzeli*, like *G.ficorum*, can also be a source of mechanical transmission of phytopatogenic bacteria and fungi (Moritz et al. 2012). *G.uzeli* originates from South-East Asia where it is widespread, but has spread to other parts of the world where *F.benjamina* has been imported. In Europe, it has been recorded in greenhouses in Germany, and subsequently in Cyprus (Collins and Philippou 2016). The species is also found in southern USA, Central and South America, Australia, New Caledonia (Mound et al. 2019), Indonesia, Hawaii and Kenya (Moritz et al. 2012).

***Haplothripsacanthoscelis* (Karny, 1910)** †

**Material examined. Malta**: Wied Għollieqa, 02.xii.2016, 4 ♀♀ (sm) on *Amaranthusviridis*, GD leg.; Msida, University of Malta grounds 02.iv.2016, 2 ♀ (sm) on *Mercurialisannua*, GD leg.; Wied Għollieqa, 16.iv.2018, 1 ♀ (sm) on *Ornithogalumarabicum*, GD leg. **Gozo**: Dwejra, 13.ix.2016, 2 ♀♀ (sm) and 3 ♂♂ (sm) on *Limoniumzerafae*, GD leg.

**Body length.** ♀: 1800–2240 µm; ♂: 1300–1560 µm.

**Wing type.** Both sexes are macropterous.

*Haplothripsacanthoscelis* is a pollen feeder and has been recorded from a number of different unrelated flowering plants (zur Strassen 1986; Trdan 2001). Such observations were also substantiated in the current study. *Haplothripsacanthoscelis* is distributed throughout Europe except the British Isles, and the East Palaearctic Region (de Jong et al. 2014).

***Haplothripsaculeatus* (Fabricius, 1803)** †

**Material examined. Malta**: Wied Qirda 06.iv.2016, 1 ♂ (sm) on *Bromusdiandrus*, GD leg.; Wied Speranza, 26.vi.2016, 1 ♂ (apterous – sm) on *Convolvuluselegantissimus*, GD leg.; Wied Ħesri, 04.xi.2016, 1 ♂ (sm) on *Cynodondactylon*, GD leg.; Wied Ħesri, 04.xii.2016, 1 ♀ (sm) on *Hypericumtriquetrifolium*, GD leg.; Siġġiewi (private garden), 02.xi.2017, 1 ♂ (sm) on *Rosa* sp., GD leg.

**Body length.** ♀: 1800–2300 µm: ♂: 1300–2040 µm.

**Wing type.** Both sexes are macropterous, though one male specimen from Malta was apterous.

This species has been recorded on a large number of unrelated plants, mainly Cyperaceae, Juncaceae and Poaceae, (Mound et al. 1976; zur Strassen 1986; Trdan 2001; Raspudić et al. 2009; Kucharczyk et al. 2011; Tunç et al. 2012; Badieritakis et al. 2015) where it feeds on pollen. In the Maltese Islands, this species was found on a number of grasses including *Bromusdiandrus*, *Cynodondactylon* but also other plant species including *Convolvuluselegantissimus* (Convolvulaceae), *Hypericumtriquetrifolium* (Hypericaceae), *Mercurialisannua* (Euphorbiaceae) and *Rosa* sp. (Rosaceae). *Haplothripsaculeatus* is preyed upon by the terebrantian thrips *Aeolothripsintermedius* (Trdan et al. 2005) and is widespread in Europe and its distribution extends to Japan (Marullo and De Grazia 2013; Minaei and Mound 2015), the East Palaearctic, Nearctic and Oriental regions (de Jong et al. 2014). This species has been intercepted in the US on plants originating from Europe (Nickle 2003).

***Haplothripssetiger* Priesner, 1921**†

**Material examined. Malta**: Dingli Cliffs, 11.iv.2016, 3 ♀♀ (sm) on *Glebioniscoronaria*, GD leg.; Dingli Cliffs, 24.v.2016, 2 ♀♀ (sm) on *Glebioniscoronaria*, GD leg. **Gozo**: Qbajjar, 31.iii.2018, 1 ♀ (sm) and 1 ♂ (sm) on *Helychrysummelitense*, GD leg.

**Body length.** ♀: 2060–2380 µm; ♂: 2040 µm.

**Wing type.** Both sexes are macropterous.

*Haplothripssetiger* is a pollen feeder which occurs on flowers of a number of deciduous plants, particularly Asteraceae (Mound et al. 1976; zur Strassen 1986; Trdan 2001; Raspudić et al. 2009; zur Strassen and Kuslitzky 2012) with a preference for dry habitats (Mound et al. 1976). In the Maltese Islands adults and larvae were found on *Glebioniscoronaria* (Asteraceae) suggesting that this plant is definitely a host of this species. Adults were also found on *Helychrysummelitense* (Asteraceae). *Haplothripssetiger* has been described as an important pollinator of *Arctostaphyllosuva-ursi* (Ericaceae) (Garcia-Fayos and Goldarazena 2008). This species has been recorded in Europe (Mound et al. 1976; Moritz 2006), the East Palaearctic Region and North Africa (de Jong et al. 2014). It has also been intercepted in the US on imported plants from Europe (Nickle 2003).

***Haplothripstritici* (Kurdjimov, 1912)** †

**Material examined. Malta**: Wied Qirda, 31.v.2016, 1 ♀ (sm) on *Hyparrheniahirta*, GD leg.; Pembroke, 05.xi.2018, 5 ♀♀ (sm, aga) on *Reichardiapicroides*, GD leg.

**Body length.** ♀: 1760–2500 µm; ♂: no records.

**Wing type.** ♀: macropterous; ♂: no records.

Another pollen feeder which has been recorded from a number of cereals and grasses (Priesner 1960; zur Strassen 1986; Mound and Teulon 1995; Raspudić et al. 2009; Kucharczyk et al. 2011; Tunç et al. 2012), but also on *Euphorbia* sp. (Euphorbiaceae), *Quercus* sp. (Fagaceae) (Tunç et al. 2012) and *Matricariachamomilla* (Asteraceae) (Raspudić et al. 2009). In the Maltese Islands, this species was found on *Hyparrheniahirta* (Poaceae) and on *Reichardiapicroides* (Asteraceae). *Haplothripstritici* has also been recorded as a prey species for the terebrantian thrips *Aeolothripsintermedius* (Trdan et al. 2005). *Haplothripstritici* has been described as a cereal pest in Europe (Priesner 1960, 1964; Mound and Teulon 1995). The distribution of this species includes Europe, extending southwards to North Africa (de Jong et al. 2014). This species is widespread in Europe. Its distribution also extends through Turkey, Iran and Iraq (Minaei and Mound 2015). *Haplothripstritici* also occurs in the East Palaearctic as well in North Africa (de Jong et al. 2014). It has also been intercepted from plant material imported in the US from the Mediterranean (Nickle 2003).

***Karnyothripsflavipes* (Jones, 1912)** †

**Material examined. Malta**: Għammieri, 16.xii.1996, 1 ♀ (sm) on dead Coccoidea on *Morusalba*, CF leg.; Msida, University of Malta grounds, 11.viii.2016, 4 ♀♀ (sm, aga) on fungus possibly *Erysipheeuonymi-japonici* on *Euonymusjaponicus* leaves, GD leg.; Siġġiewi (road), 30.10.2017, 4 ♀♀ (sm, aga) on fungus, possibly *Erysipheeuonymi-japonici* on *Euonymusjaponicus* leaves, GD leg.

**Body length.** ♀: 1780–2180 µm: ♂: no records.

**Wing type.** ♀: macropterous; ♂: no records.

This predatory species feeds on armoured scale insects (Hemiptera: Coccoidea) (Palmer and Mound 1991) as well as on mites (Arachnida), whiteflies (Hemiptera: Aleyrodoidea), and other thrips (Moritz et al. 2012), though it can also be found on dead branches, leaves, grasses and sometimes it is also associated with bamboo and Poaceae (Priesner 1960). In the current study, this species was collected from *Morusalba* (Moraceae), where it was found associated with scale insects. It was also collected on *Euonymusjaponicus* (Celastraceae), on leaves that were infected with the parasitic fungus *Erysipheeuonymi-japonici* (Erysiphaceae). *Karnyothripsflavipes* is a species which is found across the Mediterranean basin, the East Palaearctic, Afrotropical, Australian, Neotropical and Oriental regions (de Jong et al. 2014). It has also been intercepted with plant material imported in US from Europe (Nickle 2003).

***Liothripsoleae* (Costa, 1857)**

**Material examined. Malta**: Msida, University of Malta, 29.ix.2016, 2 ♀♀ (sm) from *Oleaeuropaea* leaf gall, GD leg.

**Body length.** 2000 µm; ♂: 1940–2800 µm.

**Wing type.** Both sexes are macropterous.

This species is associated with *Oleaeuropaea* (Oleaceae), where it also overwinters in cracks in the bark and reproduces in spring (Marullo 2003; Marullo and Vono 2019). *Liothripsoleae* has been described as a pest on olive trees (Priesner 1960; Marullo 2003; Marullo and Vono 2019) where it causes the formation of leaf galls. In the Maltese Islands, this species was also collected from *Oleaeuropaea*. Despite *L.oleae* being common in the Islands, and despite the deformities it causes on leaves, “… it does not cause damage of economic significance.” (Haber and Mifsud 2007: 146). Similar trends for this species were observed in Spain (Goldarazena, pers. obs.). A number of parasitoids, namely *Adelgimyzatripidiperda* (Diptera: Cecidomyiidae), *Tetrastichusgentilei* (Lewis 1997) and *Entedonastichusgaussi* (Loomans et al. 1997) (both Hymenoptera: Eulophidae) have been described on this species from Italy and Central Europe and so may be likely recorded on this species in the Maltese Islands. *Liothripsoleae* is widespread in the Mediterranean Region (de Jong et al. 2014).

***Liothripsreuteri* (Bagnall, 1913)** †

**Material examined. Gozo**: Ramla Bay, 14.viii.2017, 5 ♀♀ (sm, aga) and 2 ♂♂ (sm) on *Tamarixafricana*, GD leg.

**Body length.** ♀: 2600 µm; ♂: 2120–2380 µm.

**Wing type.** Both sexes are macropterous.

All specimens of this species collected from the Maltese Islands were macropterous, however micropterous individuals have also been described (Minaei and Mound 2015). *Liothripsreuteri* is found on *Tamarixafricana* (Tamaricaceae) (Priesner 1960, 1964; De Marzo and Ravazzi 2016) but also on *Juncus* sp. (Juncaceae) (Jenser 1982). In the Maltese Islands, adults of this species have been collected from leaves and twigs of *Tamarixafricana*. *Liothripsreuteri* is widespread in southern Europe (Marullo 1999) and across the Mediterranean basin, India and Yemen (De Marzo and Ravazzi 2016).

***Neoheegeriadalmatica* Schmutz, 1909**†

**Material examined. Malta**: Wied Ħesri, 15.iv.2016, 9 ♀♀ (sm) on *Phlomisfruticosa*, GD leg.; Wied Ħesri, 20.iv.2017, 2 ♀♀ (sm) and 1 ♂ (sm) on *Phlomisfruticosa*, GD leg.; Wied Ħesri, 03.04.2018, 6 ♀♀ (sm) and 1 ♂ (sm) on *Phlomisfruticosa*, GD leg.; Għaxqet l-Għajn l/o Naxxar 28.04.2018, 4 ♀♀ (sm) and 1 ♂ (sm) on *Phlomisfruticosa*, GD leg.

**Body length.** ♀: 2400–3400 µm; ♂: 2760–2800 µm.

**Wing type.** Both sexes are macropterous.

This species feeds on pollen and is found on Lamicaeae (Marullo and De Grazia 2013). In southern Europe, this species occurs on flowers of *Phlomisfruticosa* which belongs to the aforementioned family (Priesner 1960). This is also the plant from where both adults and larvae of this species were collected in the Maltese Islands, suggesting that this plant is definitely a host of this species. *Neoheegeriadalmatica* is distributed in south-eastern Europe (Priesner 1960), Algeria (Goldarazena, pers. obs.) and the Near East (de Jong et al. 2014).

## ﻿Discussion

Thirteen tubuliferan thrips species have been recorded in the current study. This result compares well to other islands in the Mediterranean such as Sardinia, with nine, and Crete with seven tubuliferan species (de Jong et al. 2014). Other islands, such as Sicily, with 21 (de Jong et al. 2014) and the Canary Islands, with 40 species (Berzosa 2000) show a considerably larger thrips biodiversity, probably due to the fact that these islands and archipelagos are larger and with a much wider range of different habitats that can potentially support different species, particularly the mycophagous ones.

Three species, all belonging to the subfamily Idolothripinae, are mycophagous. The remaining ten are phytophagous, with *G.ficorum*, *G.uzeli* and *L.oleae* inducing leaf galls. The remaining phytophagous species, with the exception of *Liothripsreuteri* (which was found on leaves and twigs) are pollen feeders. *Karnyothripsflavipes* is the only predatory tubuliferan species recorded in the Maltese Islands.

Analysis of chorological data based on chorotypes as defined by Vigna Taglianti et al. (1999) (Table 1, Appendix 1) showed *Gynaikothripsficorum*, and *Karnyothripsflavipes* can be described as subcosmopolitan species, although in northern countries, these have only been recorded in greenhouses.

**Table 1.** Proportion of chorotypes for the Tubulifera of the Maltese Islands.

Region	Number of species
Subcosmopolitan	2
Palaearctic	2
Mediterranean	2
Sibero European	2
Turano-Europeo-Mediterranean	2
Central Asiatic European	1
Holarctic	1
Turano Mediterranean	1
**Total**	**13**

Another three species: *Haplothripsaculeatus*, *H.setiger* and *H.tritici* are distributed across the Holarctic and Palaearctic regions, while *Bolothripsdentipes*, *B.insularis*, *Haplothripsacanthoscelis*, *Liothripsoleae*, *L.reuteri*, *Neoheegeriadalmatica* and *Priesneriellamavromoustakisi* have a European and/or Mediterranean distribution. The limited amount of habitats found on the Maltese Islands may also have contributed to the fact that no endemic tubuliferan species were found.

In the Maltese Islands, *Gynaikothripsficorum* and *G.uzeli*, which originate from South-East Asia, have little horticultural importance. Although present in large numbers within the galls they produce, these two thrips species never kill the host-plant. In fact, as the weather becomes colder, thrips colonies within the galls were observed to disperse. Also, the two species which are described as agriculturally important, namely *Haplothripstritici*, which can affect cereal crops (Priesner 1960; Mound and Teulon 1995) and *Liothripsoleae*, which has been recorded to affect olive trees and crop yield in Italy (Vono et al. 2020), were not found in large enough numbers to cause considerable damage to the plants on which they occur.

A few specimens of Tubulifera collected during the present study could only be identified to genus level. These specimens belong to the genera: *Karnyothrips* (1 species) which is likely a new species to science; *Liothrips* (1 species) and *Phlaeothrips* (1 species). The latter specimen was collected from a malaise trap.

The presence of *Androthripsramachandrai*, a predatory tubuliferan is expected in the Maltese Islands. This possibility comes from literature records of *Gynaikothripsficorum* and *G.uzeli* from different Mediterranean locations (Ali 2015; Collins and Philippou 2016), which also include the presence of *A.ramachandrai* in association with both *Gynaikothrips* species.

## ﻿Additional information

### Author ORCIDs

Godwin Degabriele https://orcid.org/0009-0003-3698-1090

Adriano Cavalleri https://orcid.org/0000-0003-4163-6745

Arturo Goldarazena https://orcid.org/0000-0003-1235-6226

David Mifsud https://orcid.org/0000-0001-9562-1077

### Data availability

All of the data that support the findings of this study are available in the main text.

## ﻿Appendix 1

**Table A1.** Chorotypes for the Tubulifera of the Maltese Islands (based on data from previously published material).

Species	Region	Notes
*Bolothripsdentipes*	Sibero European	
*Bolothripsinsularis*	Turano Mediterranean	
*Priesneriellamavromoustakisi*	Mediterranean	
*Gynaikothripsficorum*	Subcosmopolitan	
*Gynaikothripsuzeli*	Central Asiatic European	
*Haplothripsacanthoscelis*	Sibero European	also Middle East
*Haplothripsaculeatus*	Holarctic	
*Haplothripssetiger*	Palaearctic	
*Haplothripstritici*	Palaearctic	
*Karnyothripsflavipes*	Subcosmopolitan	
*Liothripsoleae*	Turano-Europeo Mediterranean	
*Liothripsreuteri*	Mediterranean	also Australian Region
*Neoheegeriadalmatica*	Turano-Europeo Mediterranean	
